# Precursor B cell all induces irreversible T cell dysfunction not solely dependent on TCR signaling

**DOI:** 10.1186/2051-1426-3-S2-P45

**Published:** 2015-11-04

**Authors:** Haiying Qin, Paul Su, Chad R Burk, Terry Fry

**Affiliations:** 1 NCI, NIH, Bethesda, MD, USA; 2 Pediatric Oncology Branch, CCR, NCI, NIH, Bethesda, MD, USA

## Background

Acute lymphoblastic leukemia (ALL) is the most common childhood malignancy. With modern risk adapted multimodality therapy, the cure rates for pediatric ALL have reached approximately 90%, but leukemia remains the leading cause of cancer-related death in children and outcomes for patients who relapse have not changed substantially over recent decades. Immunotherapy with donor lymphocyte infusions for ALL has inferior responses compared to other hematologic cancer types suggestive of challenges inherent to immunologic targeting of ALL. Blockage of immune checkpoint has led to advances in treatment of solid tumors in clinic, but has not been fully studied in leukemia models.

## Methods

Using a transplantable murine model derived from E2A-PBX1 transgenic mice we studied the impact of ALL progression on T cell function *in vivo*, and also the implication of the blockage of the immune checkpoints.

## Results

Vaccination protects mice against E2a-PBX1.3 and requires T cells and NK cells. However, therapeutic vaccination after ALL challenge is ineffective even when administered early at a low disease burden. Adoptive transfer of primed T cells from immunized donors can completely eradicate the leukemia blast. Unfortunately, T cells from leukemic mice fail to protect against E2a-PBX1.3 following adoptive transfer. T cells from leukemic mice express the programmed death receptor 1 (PD1) with progressive accumulation of additional markers of T cell dysfunction including LAG-3, and Tim-3. Furthermore, the expression of PD-1 on the T cell from the tumor bearing mice is not solely dependent on the TCR signaling. T cells from the bone marrow of children with ALL also contained large numbers of PD1+ T cells. Blockade of PD1 or its ligand, PDL1, were ineffective at preventing E2aPBX1.3 progression. Finally, expression of a chimeric antigen receptor targeting CD19 in T cells from leukemic mice fails to restore function while the T cells from naïve mice can completely cure the receipts.

## Conclusions

These findings have important implications for the optimization of immunotherapy for ALL including adoptive cell therapies utilizing CAR-expressing T cells when generated from donors with leukemia.

